# Diagnosis of Left-Sided Mechanical Prosthetic Valve Thrombosis: A Pictorial Review

**DOI:** 10.3390/jpm13060967

**Published:** 2023-06-08

**Authors:** Adela Serban, Alexandra Dadarlat-Pop, Alexandru Achim, Dana Gavan, Diana Pepine, Raluca Rancea, Raluca Tomoaia

**Affiliations:** 1 Cardiology Department, Heart Institute Niculae Stăncioiu, 19–21 Motilor Street, 400001 Cluj-Napoca, Romania; 2 5th Department of Internal Medicine, Faculty of Medicine, Iuliu Haţieganu University of Medicine and Pharmacy, 8 Victor Babes Street, 400012 Cluj-Napoca, Romania; 3 Clinical Rehabilitation Hospital, 46-50 Viilor Street, 400347 Cluj-Napoca, Romania

**Keywords:** mechanical valve thrombosis, transthoracic echocardiography, transesophageal echocardiography, multidetector computer tomography, cinefluoroscopy

## Abstract

Although transcatheter valve therapy is rapidly evolving, surgical valve replacement is still required in many patients with severe left-side valve stenosis or regurgitation, the mechanical bi-leaflet heart valve being the standard prosthesis type in younger patients. Moreover, the prevalence of valvular heart disease is steadily increasing, especially in industrialized countries, and the problem of lifelong efficient anticoagulation of these patients remains fundamental, especially in the context where vitamin K antagonists continue to be the current standard of anticoagulation despite a level of oscillating anticoagulation. In this setting, avoiding prosthetic valve thrombosis after surgery is the number one objective for both the patient and the responsible physicians. Although rare, this complication is life threatening, with the sudden onset of acute cardiac failure such as acute pulmonary edema, cardiogenic shock, or sudden cardiac death and inadequate anticoagulation remaining the leading cause of prosthesis thrombosis, along with other risk factors. The availability of multimodal imaging techniques enables and encompasses to a full extent the diagnosis of mechanical valve thrombosis. The gold-standard diagnostic methods are transthoracic and transesophageal echocardiography. Moreover, 3D ultrasound has undoubted value in giving a more accurate description of the thrombus’s extension. When transthoracic and transesophageal echocardiography are uncertain, the multidetector computer tomography examination is an important complementary imaging method. Fluoroscopy is also an excellent tool for evaluating the mobility of prosthetic discs. Each method complements the other to differentiate an acute mechanical valve thrombosis from other prosthetic valve pathologies such as pannus formation or infective endocarditis and aids the physician in accurately establishing the treatment method (surgical or pharmaceutical) and its optimal timing. The aim of this pictorial review was to discuss from an imagistic perspective the mechanical prosthetic aortic and mitral valve thrombosis and to provide an overview of the essential role of non-invasive exploration in the treatment of this severe complication.

## 1. Introduction

Four million heart valve prostheses have been implanted in the last 50 years and it is estimated that the number will increase to 850,000/year by 2050 [1]. Due to population aging and the resulting rise in degenerative heart valve disease, the number of valve prostheses is increasing. In developing nations, the rheumatic etiology is still present.

When it is feasible, surgical valve replacement or repair serves as the primary option for treating severe valvular heart disease. Surgical replacement remains the gold-standard treatment for heart valve disease, mechanical valve prostheses being the best option for young patients with unrepairable mitral and aortic valves. Mechanical valves are selected due to their long durability and improvement of late survival compared to other valve types [2].

Yet, because the prosthesis-associated pathology can eventually replace the native valve disease, we cannot assume that the patient is cured following valve surgery—they become a chronic patient requiring lifelong anticoagulant treatment and medical care.

The main complications of prosthetic heart valves are thrombosis, pannus, infective endocarditis, structural valve deterioration, regurgitation (valvular and paravalvular) or valve stenosis, prosthesis–patient mismatch, and embolic and hemorrhagic events [1]. Even if thrombus and pannus both determine valve obstruction, thrombosis represents the most common cause of prosthesis dysfunction [2]. The thrombus can be nonobstructive or obstructive and may or may not cause clinical thromboembolic events [2].

Although rare, thrombus formation on a mechanical prosthetic valve is life threatening, with the sudden onset of acute cardiac failure such as acute pulmonary edema, cardiogenic shock, or sudden cardiac death and/or cerebral or peripheral embolism [2]. The onset of symptoms may be gradual due to a slowly enlarging thrombus or sudden due to the limitation of disk motion by thrombosis of the valve hinges. Inadequate anticoagulation remains the leading cause of prosthesis thrombosis, along with other risk factors.

The availability of multimodal imaging techniques enables the diagnosis of mechanical valve thrombosis. The gold standard diagnostic methods are transthoracic (TTE) and transesophageal (TEE) echocardiography. Moreover, 3D ultrasound has an undoubted value in giving a more accurate description of the thrombus extension. When TTE and TEE are uncertain, the multidetector computer tomography (MDCT) examination is an important complementary imaging method. Fluoroscopy is also an excellent tool for evaluating the mobility of the prosthetic discs [3]. All these imagistic investigations have a clinical impact, as the evaluation of mechanical prosthetic valve obstruction focuses on distinguishing between thrombus and pannus, since thrombus may be treated with anticoagulation, thrombolysis, or valve surgery (thrombectomy or repeat valve replacement), while pannus can be treated only with valve surgery. The aim of this review is to describe the contemporary role of cardiac imaging in the diagnosis and treatment of aortic or mitral mechanical valve thrombosis and to explore how different modalities are complementary and lead to a more precise and individualized therapy.

## 2. Epidemiology

The incidence of mechanical valve thrombosis (MVT) is rare, between 0.1 and 5.7% per patient/year, but represents a life-threatening complication [4]. MVT is higher in the first year following surgery with any type of prosthesis and even up to 20% in the tricuspid position. Regardless of the time elapsed since surgery, the incidence of thrombosis for mechanical mitral and aortic prostheses is 0.5–6% per patient/year and higher in the mitral position [5].

Different data have been reported in the literature, and the incidence is possibly underestimated for a number of reasons, including the prevalence of asymptomatic cases and the difficulty of determining the diagnosis. The thromboembolic event incidence, which may or may not originate on the prosthesis, is 2.5–3.7% annually [1,6].

## 3. Clinical Presentation

MVT can have various clinical scenarios depending on the thrombus’s dimensions and the hemodynamical impact of prosthesis function. The severity of symptoms highly depends on the degree of valve obstruction caused by the prosthetic valve thrombosis. Whereas nonobstructive valve thrombosis is often asymptomatic and fortuitously diagnosed, severe obstructive thrombosis is generally associated with acute heart failure and hemodynamic collapse.

Severe obstruction appears when the mobility of the prosthesis discs is reduced or even blocked due to the overlapping of the thrombus on the disc. Intermittent obstruction is observed when the thrombus blocks the disc only sometimes and nonobstruction, when the thrombus is located on the ring and does not interfere with the closure and opening of the prosthesis. On the other hand, a thrombus can interfere with the closing of the disc, leading to intraprosthetic regurgitation. Obstruction/stenosis and/or regurgitation/intraprosthetic leak frequently coexist, rendering the prosthesis both obstructive and ineffective from a hemodynamic perspective [1].

The size, location, and whether the thrombus develops suddenly or gradually all influence the intensity of symptoms. The clinical manifestation can range from the most severe form of acute heart failure such as acute pulmonary oedema or cardiogenic shock to a reduction in exercise capacity, fatigability, and dyspnoea. Sudden cardiac death can be the first manifestation of MVT and can be also encountered at the induction stage of anaesthesia for mechanical prosthesis replacement [7,8]. Obstructive and nonobstructive thrombus of the prosthesis can lead to systemic or cerebral embolism which requires emergency imaging. In this case, TEE can help in recognizing silent thrombi and prevent thromboembolic complications [2].

In MVT, on auscultation of mechanical prosthesis the closing click is muffled or even absent. New heart murmurs might also occur. In the case of regurgitation in mitral mechanical prosthesis, a high-pitched murmur is present and in aortic prosthesis, a decreasing-intensity diastolic murmur [7]. In patients with heart failure there are also signs of pulmonary or systemic congestion.

Other rare signs and symptoms are represented by fever and chills when differential diagnosis with infective endocarditis (IE) must be considered; pallor and jaundice are associated with hemolytic anemia [9].

The imaging methods that are currently available for MVT diagnosis include transthoracic and transesophageal echocardiography, cinefluoroscopy, multidetector computer tomography, and magnetic resonance imaging.

## 4. Transthoracic and Transesophageal Echocardiographic Evaluation

In comparison to echocardiograms of native valves and the functional prosthesis, TTE assessment of prosthesis dysfunction is more challenging. Due to the reverberations of the disc, the atrial side of the mitral prosthesis and the posterior side of the aortic prosthesis are difficult to visualise. However, TTE is the standard and first imaging method in the evaluation of dysfunctional cardiac prosthesis.

The initial echocardiographic measurements, obtained one month following the surgical valve replacement, indicate the baseline hemodynamic profile of the prosthesis and will be retained as a reference value for the subsequent follow-up.

### 4.1. Prosthetic Mitral Valve Obstruction

For mitral prosthesis the TTE views that are used are the parasternal long axis (PLAX), the parasternal short axis (PSAX), and the apical 3 and 4 chambers (AP3C, AP4C), each with multiple views and intermediate angulations.

A better resolution of the mitral prosthesis should be improved by aligning the ultrasound beam parallel to the opening movement of the occluder [10,11].

However, the TTE value in the diagnosis of MVT is rather moderate, with a sensitivity and specificity of approximately 75% and 57%, respectively. Initially, thrombosis on the prosthesis is suspected due to high prosthesis gradients and velocities [12].

**Figure 1:**
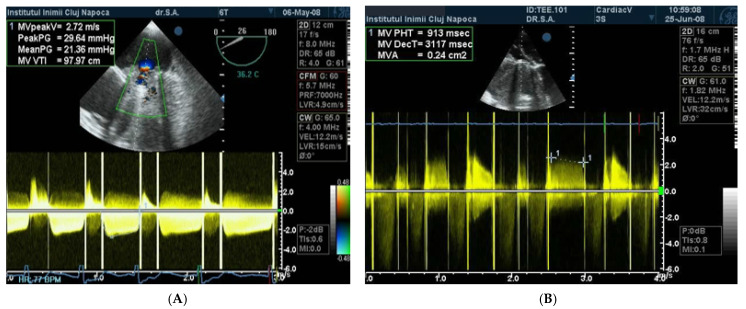
(**A**) TTE CW Doppler. Obstruction of mitral prosthesis PHT > 200 ms. (**B**) TEE CW Doppler. High gradients and velocities on mitral mechanical prosthesis are suggestive for obstruction.

The echocardiographic criteria for obstruction are obtained using continuous Doppler (CW Doppler) quantification (Figure 1); mitral prosthesis obstruction is certain if the pressure half time (PHT) value is over 200 ms, the Doppler velocity index (DVI) is over 2.2, and there is a restricted disc mobility [13].

For the diagnosis of significant prosthesis obstruction, it is recommended to use less flow-dependent parameters (Table 1) [1,13,14].

Importantly, the mean gradient is valid only under normal ventricular frequency (60–80/min). In the case of tachycardia, diastolic filling is reduced, causing a higher transprosthetic mitral gradient. The heart rate at which the pressure gradients are measured must therefore be taken into consideration [15].

For high transvalvular gradients recorded in hyper-dynamic statuses, such as fever, anaemia, and arterio-venous fistula, a differential diagnosis with MVT is required. In this circumstance, all of the valves’ recorded velocities are increased. In the case of prosthesis–patient mismatch, the transprosthetic gradients are increased for the patient’s body surface, but the prosthesis disc works normally. An abnormally high-pressure gradient is sometimes measured in the case of bileaflet mechanical prosthesis as a consequence of higher velocities registered through the central orifice, which is smaller than the lateral orifices [16].

This phenomenon can underestimate the effective area of the prosthetic orifice (EOA), leading to a misdiagnosis of prosthesis obstruction [15].

**Figure 2:**
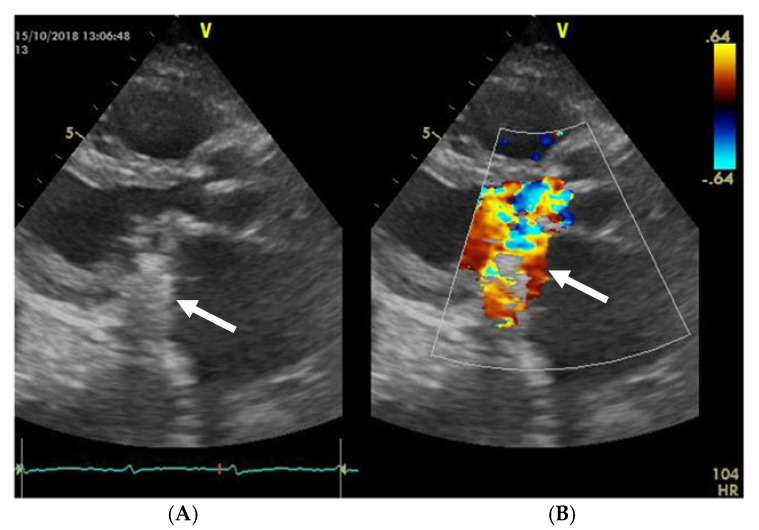
(**A**) TTE. Prosthetic mitral valve PLAX thrombus on the disc left atrial side (arrow). (**B**) TTE. Colour Doppler turbulent flow above the prosthesis (arrow).

The mobility of the discs and the presence of pathological features such as thrombus, pannus, or vegetation are evaluated using bidimensional echocardiography. The thrombus can sometimes be difficult to visualize; in some cases, it appears as an echodense mass that is located on the discs and restricts their motion (Figure 2A) [17]. The colour Doppler flow quantification may reveal a turbulent flow above the valve or even the absence of colour flow through the prosthesis (Figure 2B). While 2D imaging leaves room for ambiguity in terms of thrombus localization in relation to the prosthesis, 3D images clearly reveal the relationship between them, as illustrated in the moving images of Video S1 (Supplementary Material). The evaluation of intracardiac masses suspected for thrombi using pulsed wave (PW) tissue Doppler imaging (TDI) might be an improvement over visual assessment, as it provides a more precise definition of mass mobility [18,19].

The differential diagnosis of thrombus with pannus and vegetation is challenging and requires clinical context (Table 2). The echo density of a mass can be evaluated using the intensity ratio (defined as intensity of mass/intensity of prosthesis). A low-intensity mass, with a ratio <0.45, suggests the diagnosis of thrombosis [10,20].

**Figure 3:**
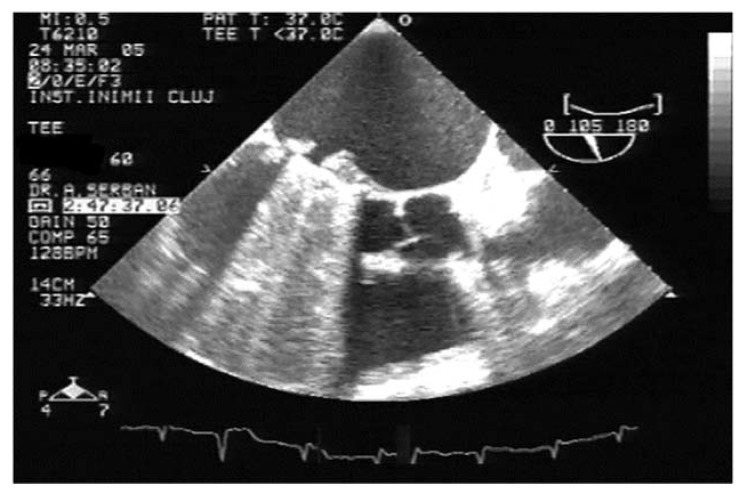
TEE. Obstructive pannus with high echo density on mechanical mitral valve prosthesis.

A pannus, in contrast to a thrombus, is a fixed mass with high echogenicity, comparable to that of the prosthetic ring (Figure 3). With the hyperplasia of connective tissue, it progresses from the ring to the prosthesis’ centre. It may also extend to the level of the hinges connecting the discs and the ring. Multidetector computer tomography (MDCT) is the method of choice to distinguish between thrombus and pannus [21,22]. For example, the degree of attenuation for the pannus has been consistently found to begreater than 150–200 Hounsfield Units (HU), while blood or even chronical thrombi have lower HU values [21].

**Figure 4:**
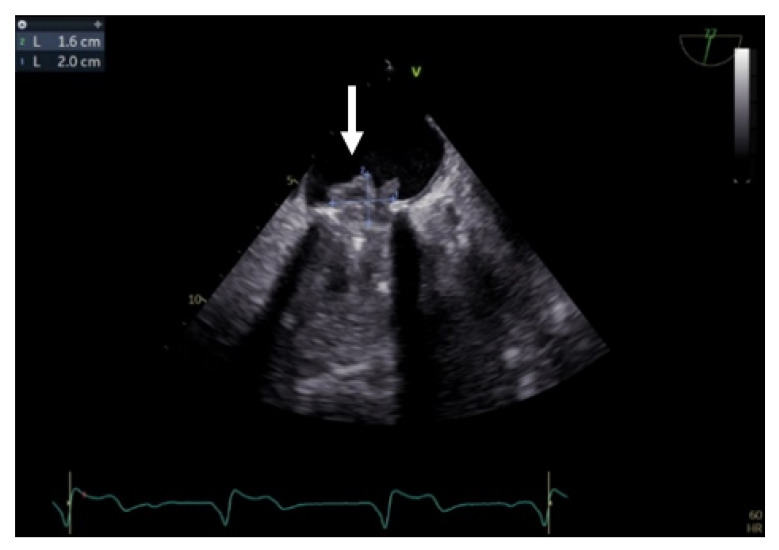
TEE. Thrombus on the atrial side of mechanical mitral prosthesis (arrow).

TEE is the gold-standard imaging method for MVT diagnosis, especially when mechanical prosthesis obstruction is caused by thrombosis [1,9,23,24,25,26]. Due to the proximity of the esophagus to the left atrium, the atrial face of any type of mitral prosthesis is accessible (Figure 4, Video S2, Supplementary Material). In the presence of obstructive thrombosis, the mobility of the discs is reduced or even blocked [23,24,27].

**Figure 5:**
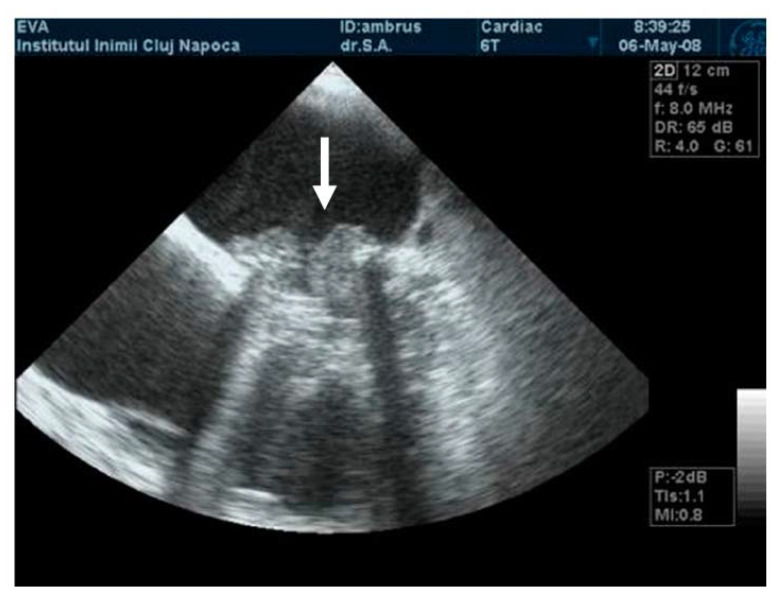
TEE. Obstructive thrombus on the atrial side of mechanical mitral prosthesis (arrow).

TEE has a major role in describing the size, mobility, and thrombus extension. It also has the ability to describe whether the thrombus is obstructive or nonobstructive, an essential aspect in the choice of therapeutic management (Figure 5) [1,21,22,25,27,28,29,30,31,32].

From the mid-esophageal four-chamber (4C) view with rotation towards 2C and 3C anteflexion and retroflexion, all the structures of the mitral prosthesis are scanned from 0 to 180 degrees. The transgastric view is necessary to visualise the ventricular side of the prosthesis, which is shadowed in the mid-esophageal view. In rare situations, the thrombus is not visualised, being located either on the hinges between the support and the disc or on the ventricular side of the prosthesis [1].

Thrombosis of the left atrial appendage or of the left atrium is important for the decision of thrombolytic treatment; isolated thrombosis of the left appendage does not preclude thrombolytic treatment [22,25,33].

**Figure 6:**
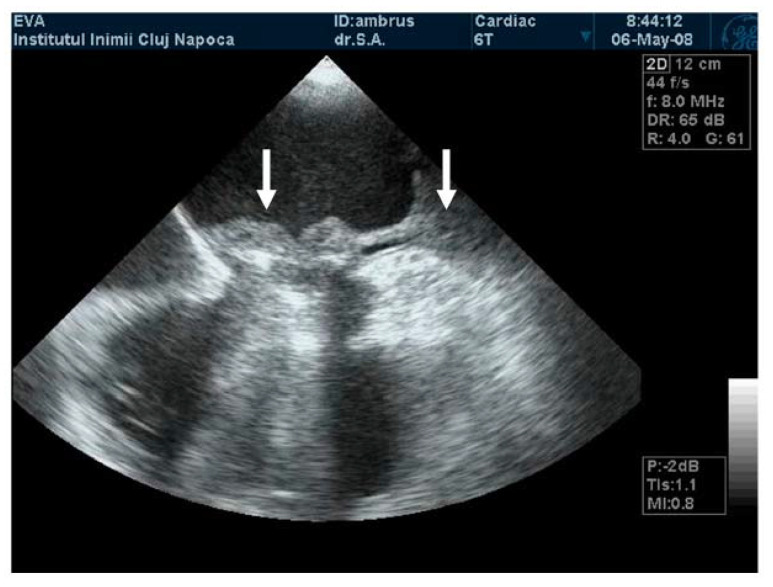
TEE. Obstructive huge thrombus on the atrial side of mechanical mitral prosthesis extended in left atrium appendix.

The thrombus can be viewed as a polylobulated, amorphous mass with an echo density equal to the myocardium, an irregular contour, and variable movement that restricts the mobility of the prosthetic discs (Figure 6). The thrombus size defines the severity of the prosthesis obstruction. Generally, a recent onset of symptomatology with hypermobile obstructive thrombi is associated with hemodynamic instability and high embolic risk [21,22,23,24]. Tong et al. reported that a thrombus on left heart prosthesis with an area below 0.8 cm^2^ is associated with a lower risk of death, as well as embolic events after thrombolytic treatment [34].

Moreover, Ozkan M. et al. reported that a thrombus area above 0.9 cm^2^ that generates obstruction of the left heart prostheses increases the risk of embolism or death associated with a thrombolytic treatment. Based on this, the 2014 ACC/AHA guideline recommends emergency surgical treatment when the size of the thrombus on the left heart prosthesis is over 0.8 cm^2^. Thrombolytic treatment is recommended when the size of the thrombus is below 0.8 cm^2^, with a recent onset of symptoms and NYHA class I–II [10].

**Figure 7:**
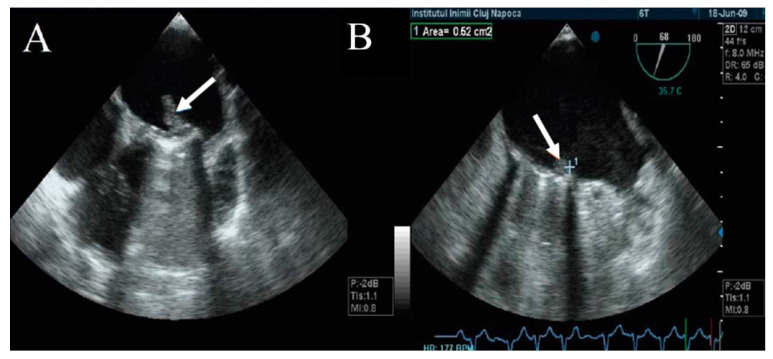
(**A**) TEE. Nonobstructive thrombus with high embolic risk (fresh, hypermobile) on the mitral prosthesis. (**B**) TEE. Small, immobile thrombus on the mitral prosthesis ring.

The embolic risk depends not only on the thrombus area, but also on the mobility and echogenicity of the thrombus. A recent onset of symptomatology and hypermobile thrombi with reduced echogenicity have a higher embolic risk than those that are hyperechogenic with reduced mobility (Figure 7) [35]. Moving images of floating thrombi on mitral prostheses are illustrated in Video S3 (Supplementary Material).

Three-dimensional real-time echocardiography (RT3DTEE) brings new perspectives in the evaluation of thrombosis on cardiac prostheses because of its superior imaging quality [36,37,38,39].

When accessible, 3D TEE is advised because it might identify prosthetic thrombi that 2D TEE misses or only partially detects [36].

The ability to identify thrombosis is improved by 3D en face reconstruction of the prostheses.

In comparison to 2D TEE, RT3DTEE provides a more accurate visualisation of the mechanical valve structures such as leaflets, rings, and struts. Leaflet restriction, softer echo density, higher mass, and quick gradient development across the valve are variables that favour thrombus over pannus. Three-dimensional TEE can also section the echogenic mass and visualize it from multiple angles, allowing the presence, extension, and localization of thrombus, pannus, or vegetation to be distinguished [40].

**Figure 8:**
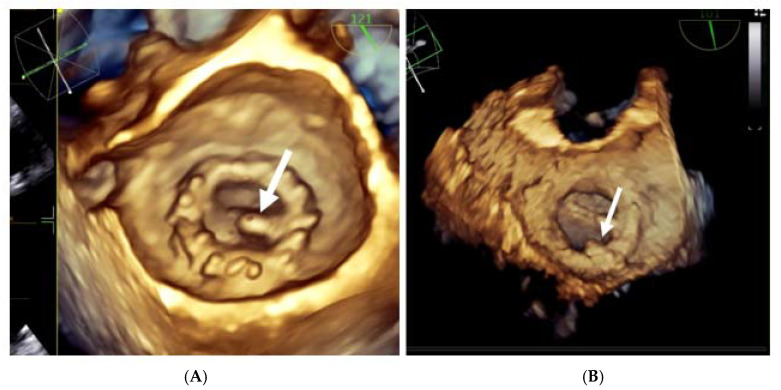
3D TEE with en face reconstruction of the mechanical mitral prosthesis with thrombus. (**A**) On disc (arrow). (**B**) On ring (arrow).

Moreover, 3D acquisitions can assist with the detection of silent thrombi that are present at the level of the prosthesis’ ring (Figure 8, panel B). In nonobstructive thrombi, where it is required to intensify anticoagulation and conduct rigorous monitoring, 3D TEE has a higher diagnostic value than 2D TEE [23,24,25,36,37,38,39].

In a study conducted on 265 patients, M. Sari et al. analysed the relationship between the location of the thrombus described in 3DTEE and the embolic risk depending on the type of prosthesis. Different prosthetic valves had significantly distinct thrombus localization patterns: type-1, involving the entire mitral annulus for Carbomedics and Sorin; type-2, involving the perihinge region and extending through some part of the annulus for St Jude Medical; and type-3, involving the mitral annulus without involving the hinge region for Medtronic ATS valves. Furthermore, prosthetic thrombosis involving only the perihinge area (type-4) was most common in patients with St Jude Medical valves (18%) [41]. The most common prosthetic thrombus localization pattern in patients with thromboembolism was type-1 (53%). Thromboembolic events were linked to older age, low international normalized ratio at admission, thrombus with a movable component >2 mm, and type-1 and type-4 thrombus localization [41].

The 3D TEE image quality depends on the accuracy of the initial 2D TEE image. A recent meta-analysis of 229 patients and 117 publications that included patients with valve prosthesis showed that 3D TEE has a sensitivity of 81% in the diagnosis of prosthesis obstruction compared to 20% for TTE [42].

Additionally, 3D TEE has limitations such as a decreased temporal resolution, poor visualization of anterior structures such as the aortic and tricuspid valves, difficulties reconstructing during atrial fibrillation, and challenges in the detection of thrombi on the ventricular side of the prosthesis [1,24,43,44].

### 4.2. Prosthetic Aortic Valve Obstruction

Similar to the mitral prostheses, TTE evaluation represents the initial diagnostic method for aortic prosthesis dysfunction through thrombosis. The diagnosis is made with the visualisation of a thrombotic mass that is more typically seen in PLAX and PSAX views on the ventricular side of aortic prostheses, and also with a restriction in the disc’s mobility. It is recommended to adjust the image for thrombus visualisation and measuring its diameter in the LVOT [23,24].

However, due to reverberations, the image is often inappropriate with posterior attenuation of aortic prosthesis. Colour Doppler imaging indicates the existence of intra- or paraprosthetic regurgitation, and PSAX incidence is used to visualize, localize, and trace leaks in accordance with a clock’s representation of the hours [13].

**Figure 9:**
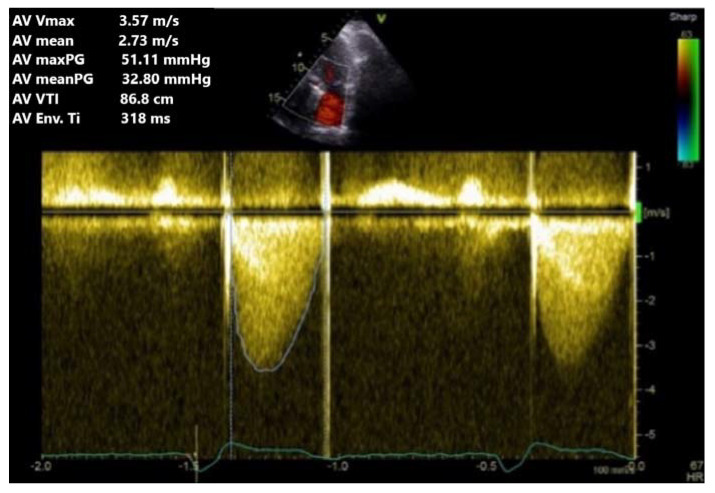
TTE CW Doppler. Obstruction of aortic prosthesis with moderate maximum and medium gradients and velocity.

Subsequently, by using apical three- and five-chamber views, continuous Doppler interrogation (CW) of the aortic prosthesis is conducted using a Doppler sample passing as parallel to the colour Doppler flow as possible (Figure 9). Transprosthetic gradients, maximum velocity, acceleration time, ejection time, and velocity time integral (VTI) in LVOT are the aortic prosthesis parameters used as a baseline for aortic mechanical valve evaluation (Table 3).

In addition, an increase in the mean gradient by more than 50% compared to the initial value or an increase in the absolute value of the mean gradient by more than 10 mmHg is considered pathological if there is no hyperdynamic status to explain these values [8].

The acceleration time is indexed to the ejection time, due to the dependence on the heart rate. The pathological values for prosthesis obstruction are over 100 ms and for the indexed-to-ejection fraction time, more than 0.37. Both DVI and acceleration time are parameters less dependent on flow and can also be used in the presence of concomitant aortic regurgitation [18].

Despite the recognition that TEE is very effective in assessing mitral prostheses, it possesses limitations with regard to aortic prostheses due to the anterior position and acoustic shadowing of the prosthetic material, especially in bileaflet valves and when there is also a mechanical valve in the mitral position. The posterior part of the aortic prosthesis is well visualized, while the anterior part is shadowed by the reverberations determined by the disc. It can be challenging to see the thrombus in some situations, requiring the use of complementary imaging techniques such cinefluoroscopy and cardiac CT [24].

**Figure 10:**
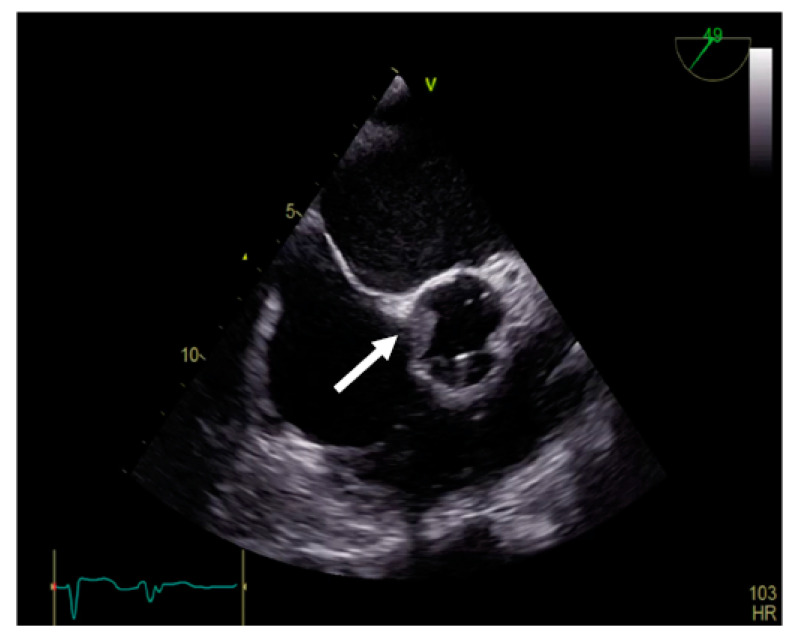
TEE. Nonobstructive thrombus on the aortic prosthesis ring (arrow).

Transgastric and deep transgastric TEE views are helpful for measuring the transprosthetic gradients and LVOT diameter in order to calculate the effective area of the aortic prosthesis. Useful information is also obtained from the mid-esophageal view at 80°, where the prosthesis is visualised in a short-axis view (Figure 10). The mobility of the discs, the symmetry of their closing–opening, and the evaluation of intra- or paraprosthetic leaks are described [13]. Stress echocardiography could be beneficial in cases of uncertain diagnosis, where the assessment of the increased transprosthetic gradient is correlated with symptom reproduction. A significant increase in the gradient through the prosthesis suggests obstruction of the aortic prosthesis [13].

## 5. Fluoroscopy

Cinefluoroscopy (CF) is a non-invasive, widely available approach to identifying prosthetic valve thrombosis, particularly for patients with bidisc prosthetic valves. The discs may be seen directly, and a tangential view can be used to measure the opening and closing angles. Prosthetic valve thrombosis is characterised by decreased leaflet mobility; these changes mostly depend on how much the prosthetic valve is blocked [23]. Video S4 (Supplementary Material) suggestively demonstrates two cases of prosthetic aortic and mitral thrombosis where indirect signs of valve dysfunction can be measured on fluoroscopic captures.

While fluoroscopy offers the most accurate evaluation of mechanical leaflet motion, it does not allow for the evaluation of soft tissue (such as thrombus or pannus) connected to the valve.

Due to significant artefacts on MDCT, it is preferable to use fluoroscopy or TEE to observe the motion of the leaflets in Bjork–Shiley and Sorin monoleaflet valves [43].

According to Montorsi et al., the sensitivity, specificity, positive and negative predictive values were 87%, 78%, 80%, and 91%, respectively for the diagnosis of prosthetic valve thrombosis in the mitral or aortic position with fluoroscopy [29].

Thrombolytic treatment (TT) has recently been adopted as the initial therapy strategy for the management of prosthetic valve thrombosis. The efficiency of TT has constantly been monitored using TEE. The use of TT for prosthetic valve thrombosis does, however, necessitate a non-invasive technique that may be used repeatedly over time to evaluate therapeutic efficacy. The pressure gradient may be restored in some patients after TT infusion, but they may still exhibit concomitant abnormal leaflet motion at CF, indicating only a partial removal of valve obstruction. The thrombus that is still there might serve as the catalyst for a late thrombotic process if lytic infusion is terminated at this point [22].

## 6. Multidetector Computer Tomography Evaluation

Although TEE is considered the gold standard in the diagnosis of MVT, the use of MDCT has become more frequent, being used especially when the diagnosis of obstruction is uncertain. The choice between TEE and MDCT as the next imaging test after TTE is made based upon availability, prior test results, patient characteristics, mechanical valve location, and operator experience [3].

For establishing the differential diagnosis between thrombus and pannus, MDCT uses the attenuation value to differentiate between these two entities and therefore influences the outcome of thrombolytic therapy in MVT, which is of significant therapeutic significance [1,19,26,44]. The CT should be conducted on a CT scanner with a high spatial and temporal resolution with retrospective acquisition and no dose modulation; the prosthesis can be evaluated in motion and on different sections (including “surgical view”), and impaired leaflet motion and coaptation can be diagnosed on the multiplanar reconstructions (Video S5, Supplementary Material).

**Figure 11:**
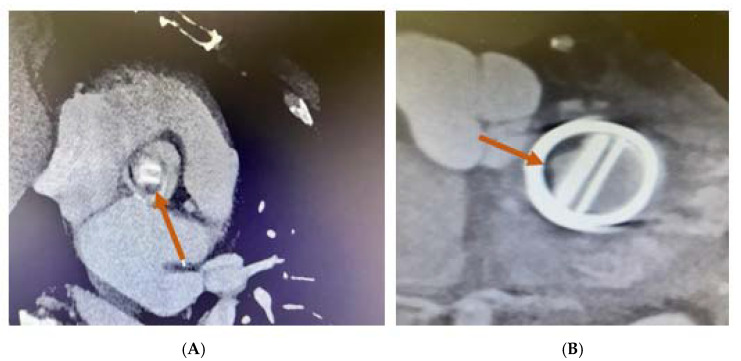
MDCT. Thrombus on (**A**) aortic mechanical valve prosthesis (arrow). (**B**) Mitral mechanical valve prosthesis (arrow).

The moment of the lesion development, as well as its location at the level of the prosthesis, are important factors in establishing its etiology. In the first year following the valve implantation, thrombus commonly develops on the aortic and left ventricular outflow tract (LVOT) sides (Figure 11).

The pannus, which is exclusively observed on the LVOT side, typically develops over a longer duration, frequently over a year [45].

In one study, Bennour et al. reported that while echocardiography was more effective in identifying thrombosis, MDCT and echocardiography yielded results for the diagnosis of mismatch that were comparable. The addition of MDCT, however, demonstrated an incremental value in the detection of pannus and leaflet restriction, which was positively correlated with thrombus [46].

MDCT appears to be the most sensitive and specific method for evaluating MVT, as it can provide anatomical and functional information, assisting in the early detection of valve thrombosis even in the subclinical stages (leaflet abnormalities) [47,48].

According to a recent meta-analysis, MDCT had the highest sensitivity (88%) for detecting subprosthetic masses. MDCT provided higher diagnostic accuracy in determining the cause of prosthetic valvular obstruction in comparison to TEE (better specificity for thrombus and higher sensitivity for pannus). In addition, it has been proven that the quality of the MDCT image is less affected by the metal components of the prosthesis, as is the case with TTE/TEE. MDCT can also analyse valve mobility, together with cinefluoroscopy being able to identify the cusp with reduced movement [42].

**Figure 12:**
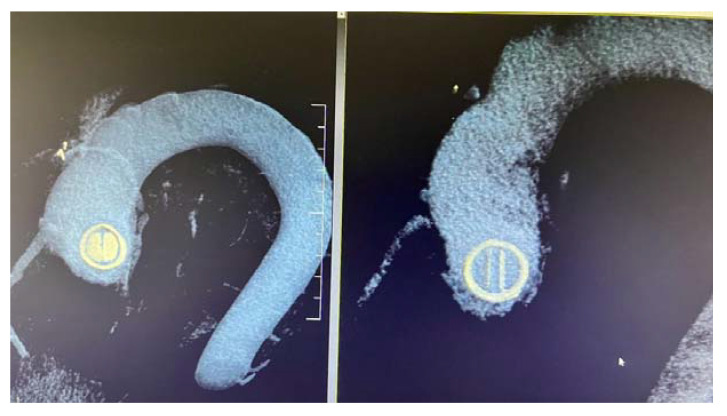
MDCT. Aortic bidisc mechanical prosthesis in diastole and systole.

In the case of mechanical MVT, MDCT facilitates the evaluation of the valve’s opening and closing angles (Figure 12) and the characterisation of masses attached to the prosthesis and the surrounding tissues [45].

It should be taken into account that MDCT also has some disadvantages in the evaluation of valve prostheses. The quality of the image is reduced in the conditions of an accelerated or irregular heart rhythm. Prosthetic valves that have cobalt–chromium components can cause severe metallic artifacts and last but not least, the risk determined by radiation exposure [49].

## 7. Magnetic Resonance Imaging

Magnetic resonance imaging (MRI) may pose important challenges due to the presence of metal devices. In general, modern implantable cardiac defibrillators and pacemakers are MR-safe, yet they may be hampered by significant artifacts. MRI safety has been investigated in 105 mechanical and 59 biological prosthetic heart valves. There were no arrhythmias, clinical complaints, pain, or safety issues particularly detected in any of the participants in three studies with 0.5-, 1-, and 1.5-Tesla MRI [50].

Although prosthetic-heart-valve-related artefacts generally remained localised and MRI was proven to be safe, the structural elements of the prosthetic heart valve could not be evaluated. With excellent accordance to echocardiography, MRI allowed measurements of the annular and orifice areas in biological valves as well as the evaluation of the flow and velocity patterns in both biological and mechanical valves [51].

MRI may potentially demonstrate abnormal asymmetrical flow patterns in prosthetic heart valve obstruction, although leaflet angle measurements may not be possible [50].

## 8. Algorithmic Approach for Imagistic Evaluation for Mechanical Prosthetic Valve Obstruction

An initial algorithmic approach is necessary mainly because of therapeutic consequences: pannus requires repeat open heart surgery to remove the pannus, while thrombotic obstruction can be treated by intensifying anticoagulant treatment, thrombolytic treatment, or repeat surgery. The first step is ubiquitous: clinical evaluation and TTE. The following steps can be adapted according to the expertise of the center in question. The ESC and ACC/AHA guidelines recommend any of the diagnostic methods (TTE, TEE, cinefluoroscopy, CT) [49,52]. The ESC guidelines emphasize the value of MDCT for differentiating pannus from thrombus [49]. The ACC/AHA guidelines state that leaflet motion should be visualized with MDCT or TEE (particularly for a mitral prosthesis) or fluoroscopy (for an aortic prosthesis) [52]. The best imaging modalities for evaluating the quality and quantity of the thrombus are TEE or MDCT [52]. An algorithmic evaluation that summarizes the two guidelines in force is proposed below.

The initial assessment of patients suspected of having MVT should include a clinical evaluation of the symptoms and signs and TTE to determine whether prosthetic valve obstruction is present. Valve obstruction is defined as significant increase in transvalvular pressure gradients since the postoperative baseline TTE (and excluding the increased stroke volume as a cause of the increased transvalvular gradients). Additional testing for mechanical valve obstruction and/or thrombosis involves imaging the mechanical valve with TEE and/or MDCT, ruling out prosthetic valve endocarditis. The choice between TEE and MDCT is made according to availability and center expertise. TEE is recommended as the primary test to evaluate prosthetic leaflet motion and appearance (although some physicians prefer CT to TEE). Since monoleaflet valves (Björk–Shiley and Sorin) are not well-visualized by CT due to severe artifacts, fluoroscopy or TEE is preferred for the visualization of leaflet motion for these valve types [53]. After either TEE or CT evaluation of the valve mass (TEE according to Table 2), the intermediate results are drawn to either a thrombus or a pannus diagnosis [54]. The CT characteristics of mass consistent with thrombus include low attenuation, irregular shape, and attachment to leaflets or hinge points. When imaging is indeterminate for thrombus or pannus, management is generally as for pannus unless the clinical features are strongly suggestive of thrombus [55]. The diagnosis also depends upon the acuity and severity of the patient’s symptoms.

## 9. Conclusions

The diagnosis of prosthesis thrombosis remains difficult, especially in emergency cases with hemodynamic instability when the therapeutic decision must be taken quickly. For all the other scenarios, the multimodal imaging evaluation starting with TTE and continuing with TEE, 3D TEE, cinefluoroscopy, and MDCT finally brings the correct diagnosis and therapeutic decision. Multimodal evaluation allows accurate and precise assessment of prosthetic valve dysfunction and hemodynamics and simple algorithms can be applied to diagnose intrinsic prosthesis dysfunction. Each imagistic method has its advantages and limitations and this review discussed how a combined complementary approach has a greater clinical impact on the identification of thrombus and its differentiation from other pathological masses.

## Figures and Tables

**Table 1:** Grading mitral prosthetic valve obstruction [1].

Echocardiographic Criteria	Severe Obstruction
Qualitative parameters	
Valve structure and motion	Abnormal occluder immobile or with restricted mobility, thrombus/pannus
Semi-quantitative parameters	
Pressure half time (ms)	>200
Quantitative parameters	
Flow-independent	
Effective orifice area of the prosthesis	<1 cm^2^
Effective orifice area vs. normal reference value	<2 SD * compared to the reference value
Difference (reference EOA—measured EOA *)	>0.35 cm^2^
Doppler velocity index	>2.5
Flow-dependent	
Maximum velocity	≥2.5 m/s
Mean gradient	≥10 mmHg
Increase in mean gradient during time	>5 mmHg
Increase in mean gradient during stress echo	>12 mmHg

* SD—standard deviation, EOA—effective orifice area.

**Table 2:** Differential diagnosis of thrombus and pannus [1].

	Thrombus	Pannus
Chronology	Occurs at any time (if late, usually associated with pannus)	Minimum 12 months from surgery (usually >5 years from surgery)
Relation with anticoagulation	Strong relationship	Poor relationship
Location	More frequent mitral valve Supra/subvalvular	More frequent aortic valve Subvalvular
Morphology	Larger mass than pannus Detected at TEE Irregular mass attached to valves/hinge point Centrifugal growth Valve restriction	Small mass Undetected at TEE (Semi)circular mass involving the suture line Centripetal growth Valve restriction may be absent
Echo density	<0.4	>0.7
Impact on gradient	MV > AV *	AV > MV *
Impact on valve orifice	MV > AV *	AV > MV *
Impact on disc mobility	Yes	Maybe
MDCT attenuation value	<200 UH	>200 UH

* AV—aortic valve, MV—mitral valve, MDCT—multidetector cardiac tomography.

**Table 3:** Grading of aortic prosthetic valve obstruction [1].

Echocardiographic Criteria	Severe Obstruction
Qualitative parameters	
Valve structure and motion	Abnormal occluder immobile or with restricted mobility, thrombus/pannus
Transvalvular flow envelope	Rounded, symmetrical
Semi-quantitative parameters	
Acceleration time (ms)	>100
Acceleration time/LV ejection time ratio	>0.37
Quantitative parameters	
Flow-independent	
Effective orifice area of the prosthesis	<0.8 cm^2^
Effective orifice area vs. normal reference value	<2 SD * compared to the reference value
Difference (reference EOA—measured EOA *)	>0.35 cm^2^
Doppler velocity index	<0.25
Flow-dependent	
Maximum velocity	≥4 m/s
Mean gradient	≥35 mmHg
Increase in mean gradient during time	≥20 mmHg
Increase in mean gradient during stress echo	≥20 mmHg

* SD—standard deviation, EOA—effective orifice area.

## Data Availability

Not applicable.

## Supplementary Materials

The following supporting information can be downloaded at: https://www.mdpi.com/article/10.3390/jpm13060967/s1, all videos (S1–S5) have a correspondent and explanation within the text.

## Abbreviations

TTE, transthoracic echocardiography; TEE, transesophageal echocardiography; MDCT, multidetector computer tomography; MVT, mechanical valve thrombosis; PLAX, parasternal long axis; PSAX, parasternal short axis; AP3C, apical three-chamber; AP4C, apical four-chamber; LVOT, left ventricular outflow tract obstruction; MRI, magnetic resonance imaging.
